# Metabolic Inflammation and Cellular Immunity

**DOI:** 10.3390/cells12121615

**Published:** 2023-06-13

**Authors:** Sardar Sindhu, Rasheed Ahmad

**Affiliations:** 1 Animal & Imaging Core Facility, Dasman Diabetes Institute, Dasman 15462, Kuwait; 2 Department of Immunology & Microbiology, Dasman Diabetes Institute, Dasman 15462, Kuwait; rasheed.ahmad@dasmaninstitute.org

Metabolic and immune cell responses are intimately linked and cross-regulated. The molecular mechanisms that drive this complex crosstalk involve a myriad of factors, including cellular and organelle stresses from glucolipotoxicity; free radical imbalance, mitochondrial and endoplasmic reticulum (ER) dysfunction; unfolded protein response (UPR); ATP bioenergetics; dysregulation of autophagy and mitophagy; impairment in biosynthetic precursors; metabolic enzymes; intermediates and bioactive metabolites of the glucose, lipid, amino acid, and nucleotide metabolism; altered immune and nutrient sensors expression and signaling; post-transcriptional and -translational modifications; and perturbations in key processes of metabolic homeostasis, energy production, and epigenetic landscape remodeling. The emerging evidence from both human and animal studies supports that the systemic nutritional status and acute increases in glucose metabolic flux fueling the high anabolic rates impact the expression of circulatory cytokines, chemokines, immune cell types, and macrophage polarization M2-to-M1 shift in the metabolically stressed adipose compartment. Metabolic signatures of key immune effector cells play a decisive role in immune response regulation. Immunometabolic research has unraveled novel key mechanisms, processes, and events within the critical pathways that regulate key functions and are potential therapeutic targets for remedying associated pathologies. However, much remains to be discovered regarding dynamic links between metabolic inflammation and cellular immunity. To this end, the editors of “Cells” took the initiative to launch a Special Issue titled “Metabolic Inflammation and Cellular Immunity”, with the aim of highlighting the emerging research and the current knowledge and understanding of the potential links between metabolic inflammation, cellular immunity, and related aspects.

This Special Issue comprises 14 key articles, including 7 original research papers and 6 review articles. We herein present an overview and introduce readers to these interesting papers. IL-23 is an inflammatory cytokine with significance in metabolic inflammation. Originally reported by Kastelein and colleagues nearly 23 years ago [1], IL-23 is now recognized as a heterodimeric cytokine composed of IL-23α and IL-12β subunits, and it plays a key role in the maintenance and expansion of T-helper type 17 (Th17) cells. It is secreted by activated monocytes, macrophages, dendritic cells, innate lymphoid cells, and γδ T cells [2]. Emerging research has shown its increasing involvement in the autoimmune and inflammatory diseases; however, its role in metabolic inflammation remains elusive. In an elegant study, Kochumon et al. showed that in individuals with high levels of low-density lipoprotein cholesterol (LDL-c), elevated adipose IL-23 expression was positively associated with typical inflammatory markers including CD11c, CD68, CD86, CD127, TLR8, TLR10, IRF3, TNF-α, IL-12, IL-18, CXCL8, CCL3, CCL5, CCL15, and CCL20, but inversely associated with plasma adiponectin levels, implying that adipose IL-23 expression could be a surrogate inflammatory biomarker in high-LDL-c populations [3].

Macrophages primed by IFN-γ or IL-4 are polarized into pro-inflammatory M1 or anti-inflammatory M2 phenotypes marked by the characteristic expression of iNOS or ARG1, respectively [4]. However, the effect of IL-4 on unpolarized macrophages during infection remains unclear. To this end, Brigo et al. showed that compared to polarized bone-marrow-derived macrophages (BMDMs), IL-4 treatment of non-polarized BMDMs resulted in improved infection control, as represented by the reduced bacterial multiplication due to metabolic reprogramming of L-arginine-dependent pathways [5].

The innate immune effector cells such as monocytes, macrophages, and dendritic cells express C-type lectin receptors dectin-1 and -2. Dectin-1 activation is known to be associated with obesity, inflammation, and insulin resistance [6,7]; however, dectin-2’s role remains unclear. Haider and colleagues showed that increased dectin-2 monocyte expression in type 2 diabetes (T2D) patients was associated with HOMA-IR and HbA1c but correlated inversely with SOCS3, which is a suppressor of JAK/STAT pathway cytokines, and involved the dectin-2-Syk-NFκB signaling [8].

Regarding T2D pharmacotherapy, metformin and fluvastatin are the two most frequently prescribed drugs. The former is an antidiabetic agent that lowers hyperglycemia and improves insulin sensitivity by reducing both intestinal glucose absorption and hepatic glucose production, whereas the latter belongs to statins, the HMG-CoA reductase inhibitors that are used to treat hypercholesterolemia and prevent cardiovascular complications in T2D patients. Notably, the effect of combination therapy on the circulating innate lymphoid cells (ILCs) in prediabetics is unclear. Mxinwa and colleagues showed that caspase-3 expression was significantly upregulated in ILC1 and ILC3 cells in mice fed a high-fat diet (HFD) compared to those fed a low-fat diet (LFD) while treatment with metformin and fluvastatin (6 weeks) reduced the caspase-3 expression, implying a protective therapeutic effect against metabolic impairment and ILC apoptosis [9].

Endocrine-disrupting chemicals (EDCs) are known to induce multiple adverse effects in both endocrine and non-endocrine organs, especially in the lungs, skin, and placenta, during pregnancy [10]. Fouyet and colleagues compared the effects of nine different EDCs on three human cell models and observed the highest P2X7 receptor activation and apoptosis in the placental cells, followed by skin cells, and found no activation in the lung cells, implying that P2X7 activation and apoptosis were key mechanisms involved in EDC-associated endocrine placental and cutaneous disorders [11].

Chronic cervical spondylosis (CCS) is a chronic, progressive degenerative disorder of the cervical spine and about 80–90% of people may develop disc degeneration by the age of 50 years. Single-nucleotide polymorphisms (SNPs) in different cytokine genes have been associated with many inflammatory disorders. An interesting study by Yadav and colleagues unraveled a significant association between the C/C and G/G genotypes and the C and G alleles of IL-1β and TNF-α, respectively, suggesting a lower risk of CCS; while the frequency distribution of risk alleles (-511T) and (-308A) was higher in CCS patients compared with healthy individuals. TGF-β had significant association with CCS susceptibility, and with age and the disease chronicity [12].

This SI includes two interesting studies involving cancer patients and systemic autoimmune disorder patients. To this end, Hynne and colleagues studied dry mouth patients that were radiated after head and neck cancer (HNC) and those with primary Sjögren’s syndrome (pSS). It is noteworthy that dry mouth condition, or xerostomia, has a multifaceted etiology, and its underlying mechanisms remain elusive. From saliva metabolomics data, the authors concluded that purinergic signaling played a critical role; increased DL-3-aminoisobutyric acid levels and dysregulated amino acid metabolism were implicated in both patient types [13]. In another study, Kucuksayan et al. reported differences in metabolism of n-10 fatty acids and signaling among three breast cancer cell lines: MCF-7, MDA-MB-231, and BT-20. The authors proposed a new role for monounsaturated fatty acid sapienic acid (6c-16:1) in membrane plasticity and protein signaling, suggesting that the tailored membrane lipid strategies could have translational significance for guiding pharmacological interventions in breast cancer [14].

Among the eminent review papers published in this Special Issue, Poto and colleagues reviewed the pathogenic as well as therapeutic potential of neutrophil extracellular traps (NETs: net-like structures composed of DNA scaffolds, histones and granular proteins released by activated neutrophils) in asthma, which is consistent with their deleterious roles in autoimmunity, cancer, and allergy as well as beneficial effects via NETosis and resolution of inflammation by degrading cytokines/chemokines. The authors also elucidated putative surrogate NET biomarkers in this study [15].

The potential origin of polygenic autoinflammatory diseases (ADs) in children remains elusive. In these conditions, activation of the innate immunity leads to cytokine-mediated inflammation, without familial recurrency. Regarding the periodic fever/aphthous stomatitis/pharyngitis/cervical adenopathy (PFAPA) syndrome, Sangiorgi and Rigante reviewed and discussed the significance of multi-aspect data analyses including clinical data; inflammatory parameters at different disease phases; therapeutic efficacy of corticosteroids, colchicine, or IL-1 antagonists; and the robustness of genotypic analysis to confirm or exclude a monogenic origin of the disease [16].

Regarding adaptive immunity, presentation of a diverse range of auto-antigens in the thymus leads to the formation of T-cell repertoires that recognize self, altered-self, and non-self antigens. In their paper, Shevyrev et al. reviewed and discussed how thymic epithelial cells play a key role in the promiscuous gene expression of nearly the whole spectrum of proteins encoded in the genome. It was also pointed out that a noncanonical transcription factor called autoimmune regulator (AIRE) orchestrated this intricate mechanism. The authors highlighted the phylogenetic prerequisites for the development of modern adaptive immunity and significance of the antigen presentation system [17].

Owing to their critical roles in physiology and pathophysiology, monounsaturated hexadecenoic fatty acids, such as palmitoleic acid (16:1n-7), sapienic acid (16:1n-10), and hypogeic acid (16:1n-9), are emerging as key biomarkers of health. Thus, Bermudez and colleagues reviewed and discussed the current literature and regulatory effects of palmitoleic, sapienic, and hypogeic acids in various metabolic disorders including T2D, cardiovascular disease, non-alcoholic fatty liver disease, and cancer [18].

Importantly, extracellular vesicles (EVs) are emerging as key mediators in the pathophysiology of obesity and associated metabolic disorders. To this end, Delgadillo-Velazquez et al. reviewed relevant studies, highlighting the role of EVs in adipose tissue inflammation and metabolic adaptation via the PI3K/Akt/mTOR activation underlying the crosstalk among metabolically active organs [19].

Lastly, addressing aging and the associated genomic, epigenetic, and metabolic changes, Haupt and colleagues reviewed the current literature on biological age predictors that drive cellular senescence, such as telomere length, DNA methylation, and metabolic footprints, and discussed the impact of exercise on aging [20].

We thank all our contributors, who enriched this issue with their highly valuable and interesting studies.
